# The Open Aurignacian Project: 3D scanning and the digital preservation of the Italian Paleolithic record

**DOI:** 10.1038/s41597-025-05330-z

**Published:** 2025-06-19

**Authors:** Armando Falcucci, Adriana Moroni, Fabio Negrino, Marco Peresani, Julien Riel-Salvatore

**Affiliations:** 1 Department of Geosciences, Prehistory and Archaeological Sciences Research Unit, Eberhard Karls University of Tübingen, Tübingen, Germany; 2 Department of Physical Sciences, Earth, and Environment, Prehistory and Anthropology Research Unit, University of Siena, Siena, Italy; 3 Center for Quaternary Studies (CeSQ), Sansepolcro, Italy; 4 Italian Institute of Human Paleontology (IsIPU), Anagni, Italy; 5 Department of Antiquities, Philosophy, and History, University of Genoa, Genoa, Italy; 6 Department of Humanities, Prehistoric and Anthropological Sciences Unit, University of Ferrara, Ferrara, Italy; 7 Institute of Environmental Geology and Geoengineering, National Research Council, Milan, Italy; 8 Department of Anthropology, University of Montreal, Montreal, Canada

**Keywords:** Archaeology, History

## Abstract

Here, we introduce an open-access database of 3D models of stone tools (n = 2,016) from four Early Upper Paleolithic sequences excavated south of the Alps and along Peninsular Italy, including Grotta della Cala, Grotta di Castelcivita, Grotta di Fumane, and Riparo Bombrini. Available through four self-standing Zenodo repositories, these models enable in-depth analysis of core reduction procedures, reduction intensity, and shape variability. Unlike other repositories, this database has been actively used to address archaeological questions, providing a comprehensive demonstration of the use of 3D models in lithic analysis. The Open Aurignacian Project utilizes various scanning devices, including the Artec Spider, Artec Micro, and micro-computed tomography, with a focus on enhancing the reproducibility and accessibility of archaeological data. This paper presents the scanning methodology, dataset organization, and technical validation of the project, while also discussing the scientific potential of these data to foster cross-continental research collaboration. Our open-sharing initiative is designed to stimulate inter-regional studies of human behavioral evolution, offering new opportunities to address questions in Paleolithic studies through the FAIR principles.

## Background & Summary

### Introducing the Open Aurignacian Project

**Fig. 1:**
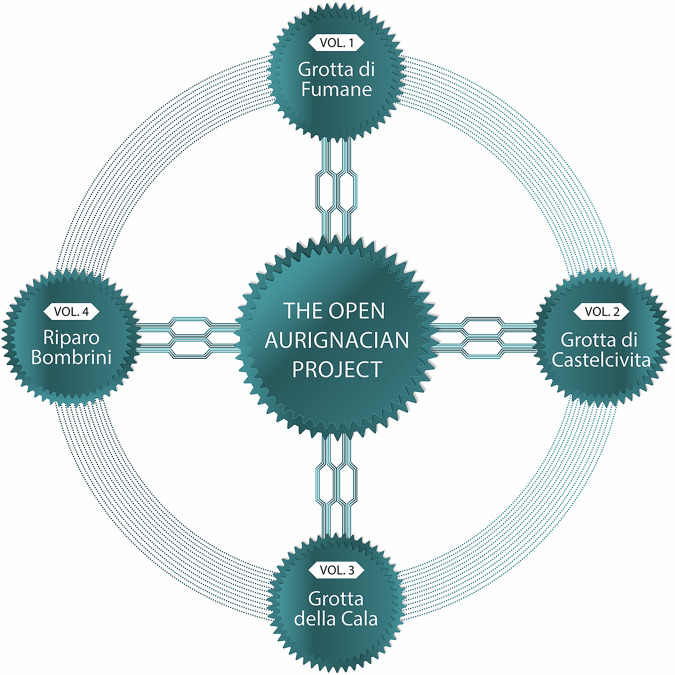
Visual organization of the Open Aurignacian Project, showing the four sites scanned so far and organized into separate Zenodo repositories (referred to here as Volumes). Volume 1: 10.5281/zenodo.6362149^4^; Volume 2: 10.5281/zenodo.10631389^1^; Volume 3: 10.5281/zenodo.14165189^2^; Volume 4: 10.5281/zenodo.14731694^3^.

The Open Aurignacian Project (OAP) aims to systematically scan and create open-access repositories of 3D meshes of stone tools from stratified sites dating to the early stages of the Upper Paleolithic south of the Alps and along Peninsular Italy, with a particular focus on the Aurignacian technocomplex. To date, the OAP has led to the publication of four distinct repositories on Zenodo^1–4^ (Fig. 1), totaling 2,016 stone tools. These 3D models were created using high-resolution 3D scanning devices, which allow for detailed and accurate digital representations of each artifact’s shape, size, and surface features. The division into four distinct repositories ensures proper attribution to the coordinators of fieldwork and research at each site. This data descriptor aims to enhance the discoverability and accessibility of these valuable datasets, which are fully open access under the Creative Commons Attribution 4.0 International license. In addition to this paper, the OAP platform—hosted at https://www.armandofalcucci.com/project/open_aurignacian/—provides updates and new releases of datasets from additional sites and/or stratigraphic units. These repositories were created with the goal of enabling FAIR (Findable, Accessible, Interoperable, and Reusable) sharing of data, supporting several published papers. More importantly, the data provides an open resource for testing novel hypotheses related to human behavior and lithic technology, fostering cross-continental collaborations, and serving as a platform for education and outreach. Through the OAP, we aim to inspire other researchers to adopt open data-sharing practices for 3D archaeological materials, maximizing the potential of this technological innovation to advance archaeological research.

### Background on 3D modelling in lithic analysis

Since its early development in the second half of the 20th century, 3D scanning technology has primarily been employed in industrial settings for applications such as quality control and reverse engineering^5^. Over time, mainly due to technological advancements, the applications of 3D scanning have expanded into various fields, including archaeology, where it has played a significant role in the last 20 years^6–8^. While the use of 3D modeling in archaeology has been intermittent, it is now regularly applied across different scales, from site-level documentation^9–11^ to the digitization of a wide range of organic and inorganic artifacts recovered during excavations^12–17^. One area in archaeology that has particularly benefitted from 3D modeling is lithic analysis. As noted by Wyatt-Spratt^18^, over 200 papers have been published as of 2022 directly utilizing 3D models for analytical and visualization purposes. This increase in use is largely driven by the increasing affordability of scanning devices and the development of open-source software tailored to analytical needs^19^.

3D models can be created using various scanning techniques, including laser scanning, structured light scanners, micro-computed tomography (micro-CT), and photogrammetry^18–22^. These techniques enable precise digital replicas of stone tools that can be analyzed and measured in ways that were previously challenging with traditional methods. The pioneering work of Riel-Salvatore, *et al*.^23^, demonstrated the potential of 3D scanners for lithic analysis, specifically for scar pattern segmentation and virtual refitting. Since then, 3D models have helped overcome challenges in lithic replicability^24^, particularly in quantifying morphological features of stone tools. A few studies have explored the use of 3D models to address inter-observer variability in lithic analysis, often through collaborative research frameworks^25,26^. Researchers have also developed new tools and open-source software for quantifying parameters that cannot be recorded manually^27–29^.

For example, several studies have used 3D geometric morphometrics (3DGM)^30^ approaches to quantify shape variability in stone tools^28,31–40^, as well as the measurement of 3D angles^41–46^. Other applications include quantifying reduction intensity^47–51^, cortex coverage on blanks and cores^52^, use-wear analysis^53–55^, ballistic analysis^56^, the 3D geometry of tool edges^57^, virtual knapping^58^, and virtual refittings^59–62^. Additionally, 3D modeling has provided critical insights into technological variability in lithic production^27,63–66^, and has been invaluable in the study of experimental flintknapping, where 3D models record the morphological and volumetric changes of cores at different stages of reduction^47,49^.

3D modeling has also facilitated the creation of digital repositories for educational and preservation purposes^67,68^. Notable examples include the DISAPALE project by the Neanderthal Museum in Germany^69^ and the Museum of Stone Tools (stonetoolsmuseum.com), directed by M. Moore, which hosts a large number of 3D models from various regions and periods. These repositories provide valuable resources for research, outreach, and teaching. Furthermore, the sharing of 3D models through open science practices enables inter-institutional collaborations and promotes more sustainable research practices by reducing the need for physical movement of artifacts and researchers^70^. Open access to 3D datasets is particularly important for fostering collaborative networks and ensuring access to lithic collections for researchers in low- and middle-income countries.

Despite the remarkable progress in creating repositories for educational and preservation purposes, these databases often fall short when it comes to hypothesis-driven research. They are typically not designed to scan complete artifact classes within specific stratigraphic layers from Paleolithic sites. On the other hand, 3D models created for analytical purposes are often not openly shared^18^, limiting their most valuable feature: the ability to access raw data, replicate analyses, and develop new research. Also, this scientific practice contradicts the paradigm shift toward open and replicable science^71,72^. However, some notable exceptions of accessible 3D databases do exist^64,73–78^. The OAP database was specifically developed to provide new open data supporting the analysis of the Italian Aurignacian, enabling innovative research that has already proven pivotal^79^.

### The Aurignacian

The Aurignacian is often regarded as the first pan-European technocomplex. Initially considered a marker for the spread of anatomically modern *Homo sapiens* across Europe^80^, it is now understood as the result of more complex biocultural processes that reached a resolution around 42,000 years ago^81–83^. This technocomplex unified a vast geographic area, extending from the northern Mediterranean Basin to the Atlantic and continental Europe^84,85^. The Aurignacian has garnered significant scientific attention due to its remarkable features, including increased evidence for symbolic behavior^86–88^, long-distance mobility practices and networks^89–91^, and the manufacture of multi-component projectiles^92–94^. The Aurignacian encompasses several techno-cultural variants, which are typically found in stratigraphic sequence, reflecting a clear chronological succession^95–98^. Lithic and bone tool data have played a significant role in defining the main stages of the Aurignacian^99–102^. However, the observed similarities and variability in these tools are driven not only by cultural developments but also by diverse land-use strategies and mobility patterns across the Aurignacian’s large geographic scope^87,89,103–106^.

In Italy, the Aurignacian is found in both stratified and open-air sites across different environmental settings^107,108^. Notably, evidence suggests that the Aurignacian began later in southern Italy, as seen at sites like Grotta di Castelcivita^82^. The OAP was developed to analyze several stratified sites across Italy, which has allowed for a better understanding of Upper Paleolithic developments in the region, particularly the role of mobility strategies and environmental variability in shaping techno-cultural diversity^31,49,95,109,110^. For example, research in southern Italy has shown that the major super-eruption of the Campanian Ignimbrite around 40,000 years ago^111^ had little or no impact on the technological development of the Aurignacian^95^. Moreover, evidence suggests human occupation continued in the region shortly after the eruption, as demonstrated by findings at Grotta della Cala^112^.

While the Italian Aurignacian was previously not central to discussions on the development of the Upper Paleolithic, recent technological studies—complemented by the large-scale digitization of the 3D models presented in this paper—have provided an unprecedented volume of data. This allows for a deeper discussion on human behavior, the role of cultural transmission, and environmental shifts in the observed cultural variability across Europe. Importantly, these renewed technological studies provide a robust foundation for utilizing the 3D models presented in this paper, facilitating the design of research projects that address meaningful archaeological inquiries.

## Methods

### Site selection

**Fig. 2:**
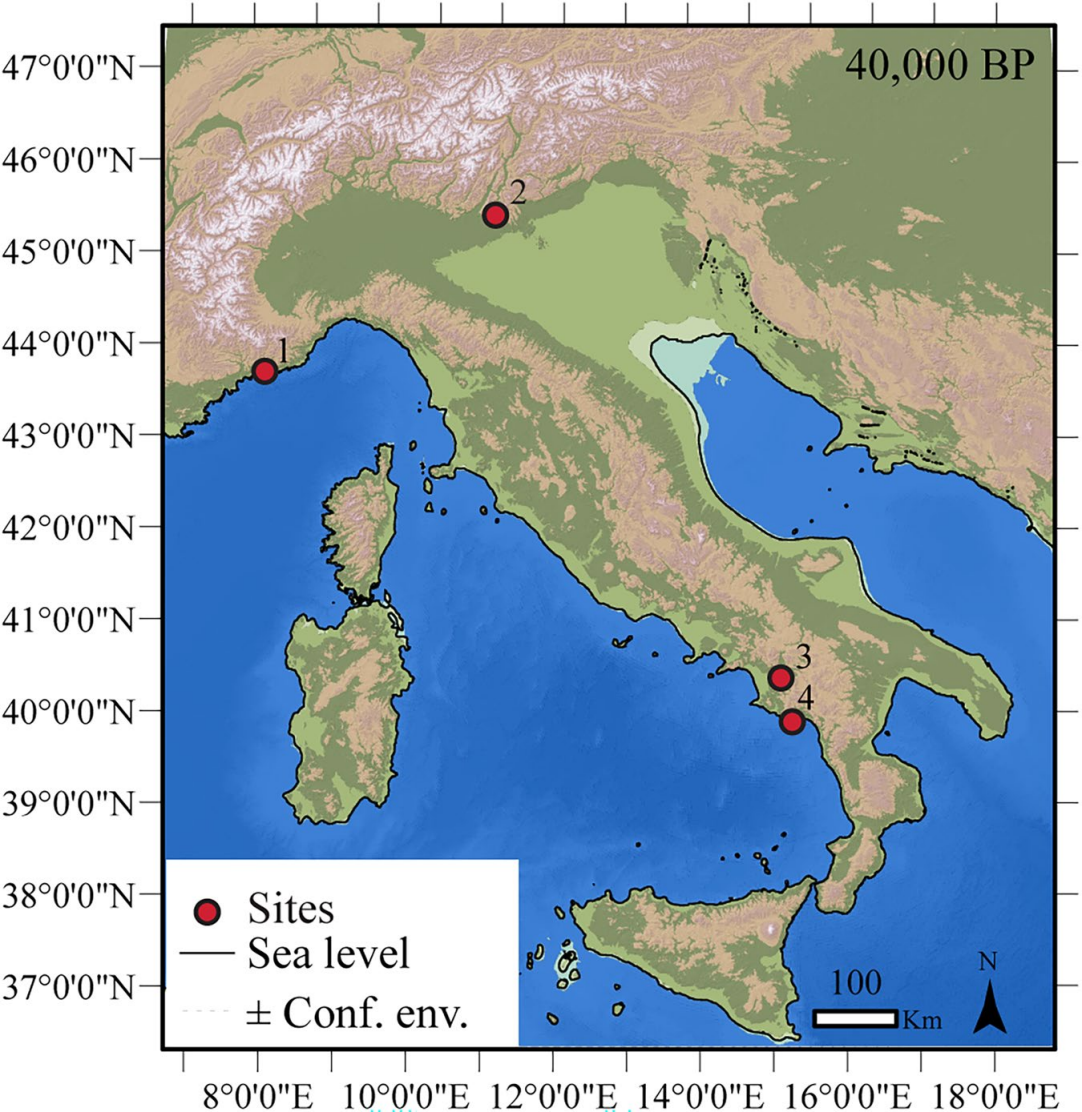
Geographic location of the sites part of the Open Aurignacian Project: (1) Riparo Bombrini, (2) Grotta di Fumane, (3) Grotta di Castelcivita, and (4) Grotta della Cala. The maps show the paleo-geographic reconstructions of Italy, taking into account mean sea-level estimations with associated confidence envelopes^148^ at about 40,000 BP (−62 ± 13 m above the current sea level). The map was generated using ArcGIS®10.8: (https://desktop.arcgis.com/en/arcmap/latest/get-started/setup/arcgis-desktop-system-requirements.html). Source of the Digital Elevation Model: GMES RDA project (https://www.eea.europa.eu/data-and-maps/data/eu-dem#tab-originaldata/eudem_hlsd_3035_europe). Source of the Bathymetry: EMODNET (https://emodnet.ec.europa.eu/en/bathymetry). Map: Courtesy of V. Spagnolo (University of Siena).

The OAP is divided into four sub-projects, each corresponding to one of the sites analyzed during the research project on the Italian Aurignacian (https://gepris.dfg.de/gepris/projekt/431809858?language = en), funded by the German Research Foundation (DFG). These sites have so far been instrumental in answering key archaeological questions. All sites are located in Italy, with two situated in the north—Grotta di Fumane^113^ and Riparo Bombrini^114,115^—and two in the south—Grotta della Cala^112,116^ and Grotta di Castelcivita^95,117^ (Fig. 2). Each sampled stratigraphic unit is associated with the Aurignacian, and ongoing research and fieldwork at all sites have ensured a high degree of control over the chronological and stratigraphic integrity of the data. Only the repository for Grotta di Fumane contains a few lithics associated with the Early Gravettian^118^. At Grotta di Fumane and Riparo Bombrini, remains identified as anatomically modern *Homo sapiens* (e.g., two teeth) were found in association with the lowermost layers, attributed to the Protoaurignacian^119^. All sites are well-known for their rich material culture, which spans lithic and bone tools, symbolic artifacts, and evidence of living features such as hearths and other spatial arrangements^109,120–123^. Table 1 provides a detailed overview of the studied sites, including information on the chronological framework, stratigraphic units, techno-cultural attribution, and current research directors. Additionally, it lists the published datasets linked to the technological analyses of the lithic assemblages.

**Table 1:** Stratigraphic data, techno-cultural attribution, chronological range, respective fieldwork directors, and published lithic datasets for the four Aurignacian sites in Italy, demonstrating the comprehensive nature of the data available for research.

Site	Stratigraphic data and techno-cultural attribution	Chronology	Director(s)	Published datasets
Grotta della Cala (n = 420)	AU10 = Early Aurignacian AU11 = Early Aurignacian AU12 = Early Aurignacian AU13 = Early Aurignacian	39.9–37.4 ky cal BP^82^	A. Moroni	^2,112,136^
Grotta di Castelcivita (n = 538)	*ars = *Early Aurignacian *gic = *Early Aurignacian *rsa’* = Protoaurignacian	41.8–39.9 ky cal BP^82^	A. Moroni	^1,95,137^
Grotta di Fumane (n = 948)	D1d, D1f = Early Gravettian D1, D1c = Aurignacian* D3b = Aurignacian* D3b alpha = Early Aurignacian D3d = Aurignacian* D3d base = Aurignacian* D3 + D6 = Aurignacian* D6 = Aurignacian* A1 = Protoaurignacian A2 = Protoaurignacian	41.2-33.2 ky cal BP^118,138,139^	M. Peresani	^4,31,109,110,140–142^
Riparo Bombrini (n = 110)	A0 = Early Aurignacian A1 = Protoaurignacian A2 = Protoaurignacian	41-2–35.9 ky cal BP^119^	F. Negrino & J. Riel-Salvatore	^3,105,143^

*A more detailed techno-cultural attribution is not possible due to stratigraphic issues.

### Data recording

A total of 2,016 lithic artifacts have been digitized in 3D using various scanning technologies (Table 2) and protocols. These scanning protocols were developed in recent years by one of us^19,22,124^. The observed numerical differences in the number of 3D models per site can be attributed to variations in the density of finds at each site, as well as the number of stratigraphic layers analyzed in this project.

**Table 2:** Distribution of 3D digitized stone tools by scanner type (i.e., Artec Micro 1, Artec Space Spider 1, and micro-CT) used across the four Aurignacian sites.

Site	Artec Micro	Artec Spider	Micro-CT	Total
Grotta della Cala	51 (12.1%)	369 (87.9%)	—	420
Grotta di Castelcivita	144 (26.8%)	394 (73.2%)	—	538
Grotta di Fumane	—	377 (39.8%)	571 (60.2%)	948
Riparo Bombrini	—	110 (100.0%)	—	110
Total	195 (9.7%)	1,250 (62.0%)	571 (28.3%)	2,016

Percentages are provided in brackets.

The majority of the 3D meshes were generated using an Artec Space Spider scanner (n = 1,250) from Artec Inc. (Luxembourg). This portable scanner is particularly effective for scanning lithic artifacts *in situ*. The Artec Spider offers accuracy of up to 0.05 mm and an ultra-high resolution of up to 0.1 mm, making it ideal for medium-sized objects. For scanning, a turntable was used, capturing two or three views of each lithic artifact before running the data through standard algorithms in Artec Professional Software. Although the Artec Spider is also suitable for scanning small-sized artifacts, the scanning resolution diminishes for thin objects with a maximal length of ca. 3 cm and sharper edges^19^. Therefore, to scan a large number of small artifacts, we also used micro-CT technology (n = 571) following the *StyroStone* protocol^19,124^ and an Artec Micro scanner (n = 195), following the *MicroStone* protocol^22^. The Artec Micro has an accuracy of up to 0.01 mm and a resolution of up to 0.029 mm, making it ideal for capturing fine details on smaller objects. The micro-CT scanner (Phoenix v-tome-x s model by General Electronics MCC, Boston, MA) was used to scan several hundred lithics from Grotta di Fumane, with the primary aim of conducting 3DGM analysis of blade and bladelet implements^31,110^.

**Fig. 3:**
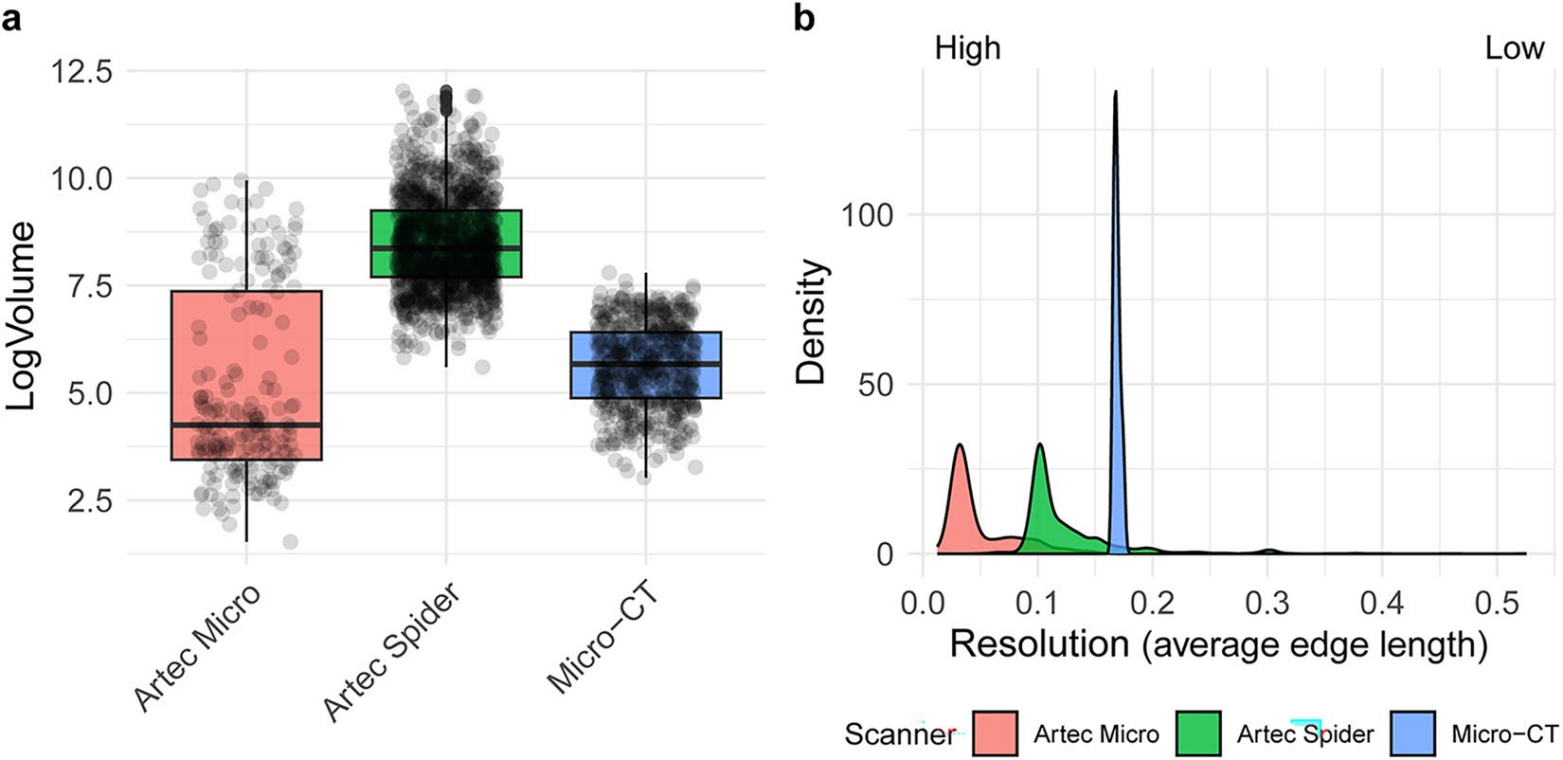
(**a**) Boxplots with jitter points showing the comparison of volume between the three scanning devices. Due to the large values in the Artec Spider group, which masked variability in the lower range, the volume was log-transformed to improve the visibility of the data distribution and better highlight the differences. (**b**) Density distributions of average edge length (in mm) between the points of the 3D model (i.e., the resolution) across the different scanners used in this project.

The *StyroStone* protocol has proven highly effective for minimizing scanning time while maximizing the number of lithics scanned in a single session. For instance, up to 220 bladelets were scanned in a single session and later extracted as individual 3D models. The resolution for these scanning sessions was set to 140 microns, providing an effective balance between productivity and model quality. Although the resolution of the smallest bladelets scanned with the micro-CT does not match that of the Artec Micro, Göldner, *et al*.^19^ validated this method by conducting a 3DGM comparison between a sample of bladelets scanned using a micro-CT scanner and an Artec Micro scanner. The micro-CT models were also utilized for quantifying 3D angles of retouching^110^. Also, while the Artec Micro is better suited for capturing features such as lateral retouching, even on bladelets as small as 1 cm, it requires more time to produce models. The comparison of volume values between the different scanners shows that the Artec Spider was used to scan the largest artifacts, while the Artec Micro and the micro-CT scanners were mostly used for small-sized artifacts, though the Artec Micro was occasionally employed to scan a few larger ones (Fig. 3a). We compared the resolution across different scanners by measuring the average edge length between the points of the model^125^ (Fig. 3b). Higher-resolution scans correspond to smaller average edge length values. The results clearly show that the highest resolution was achieved with the Artec Micro, while the lowest was obtained with the micro-CT scanner. To improve resolution—especially when aiming to produce accurate 3D models of small-sized artifacts—we recommend reducing the number of pieces scanned in a single micro-CT session. This allows the objects (i.e., the target) to be positioned closer to source, thereby enhancing overall resolution^19^. Furthermore, since the lithic artifacts were of similar size and the scanning distance remained constant, the resolution of the micro-CT scans was more consistent across the resulting 3D meshes compared to the other scanning devices.

### Selection of artifacts

**Fig. 4:**
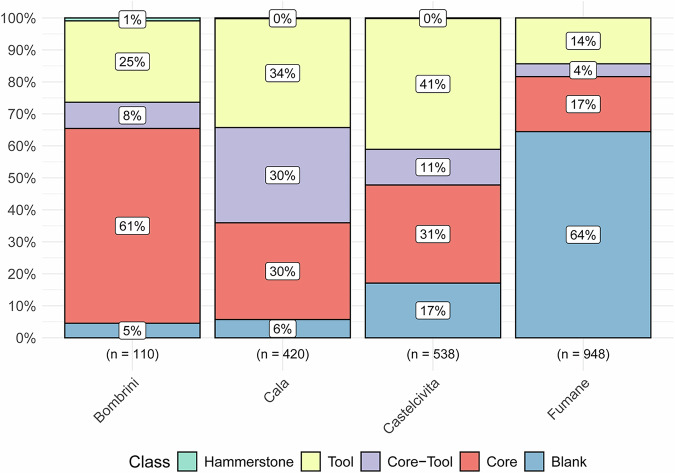
Distribution of available scans across the main lithic classes, illustrating the differences between the studied sites. The total number of scans for each site is reported in parentheses below the bars, while the percentages for each lithic class are shown inside the stacked bars.

**Fig. 5:**
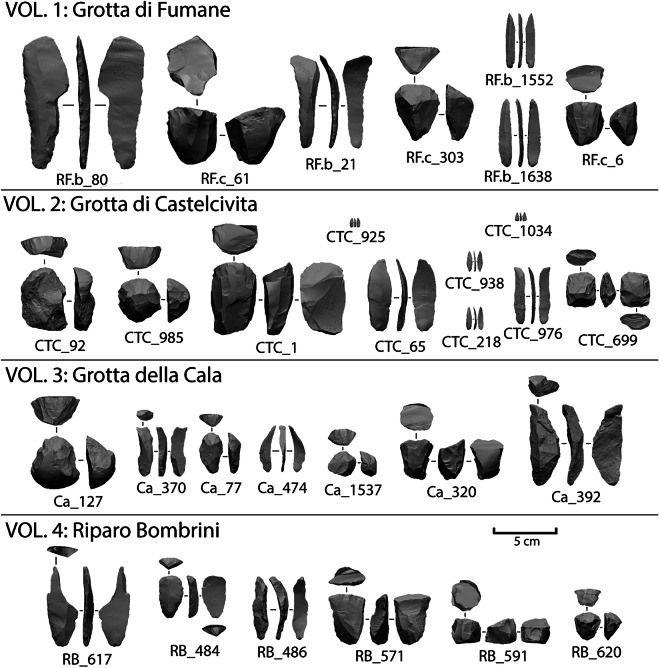
3D models of stone tools from the four sites of the Open Aurignacian Project. Categories include retouched blades and burins (RF.B_80, RF.b_21, CTC_65, Ca_370, Ca_392, RB_617), blade/let cores (RF.c_61, RF.c_303, RF.c_6, CTC_985, CTC_1, Ca_127, Ca_77, Ca_1537, Ca_320, RB_571, RB_591, RB_620), retouched bladelets (RF.b_1552, RF.b_1638, CTC_938, CTC_218, CTC_1034, CTC_976), endscrapers (CTC_82, RB_484), flake cores (CTC_699), and unretouched blade/lets (Ca_474, RB_486). The artifacts were scanned with an Artec Spider (i.e., RF.b_80, RF.c_61, RF.b_21, RF.c_303, RF.c_6, CTC_92, CTC_985, CTC_1, CTC_65, CTC_699, Ca_127, Ca_370, Ca_77, Ca_474, Ca_320, Ca_392, RB_617, RB_484, RB_486, RB_571, RB_591, RB_620), a micro-CT scanner (i.e., RF.b_1552 and RF.b_1638), and an Artec Micro (i.e., CTC_938, CTC_218, CTC_1034, CTC_976, Ca_1537). More contextual and technological information is available in the associated metadata files for each repository.

Stone tools were scanned as part of techno-typological studies aimed at investigating Aurignacian core reduction procedures, reduction intensity, and shape variability, both within and between sites. Figure 4 shows the percentage distribution of available scans across lithic classes, highlighting differences between the studied sites. At all sites, we primarily focused on scanning cores associated with blade and bladelet productions (see Fig. 5).

**Fig. 6:**
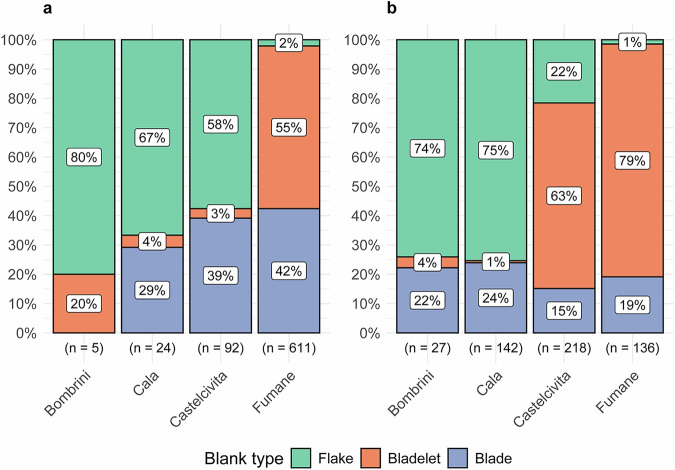
(**a**) Proportion of unmodified blanks scanned across the studied sites, highlighting differences in the frequency of blank categories. (**b**) Proportion of scanned tools, categorized by blank type, across the studied sites. The total number of blanks and tools for each site is reported in parentheses below the bars, while the percentages for each lithic class are shown inside the stacked bars.

At Grotta della Cala, Grotta di Castelcivita, and Riparo Bombrini, all cores related to flake production were also scanned. Regarding blank types, the frequency differences are linked to the scanning technologies used, which influenced the ability to digitize small-sized bladelets. Flakes are the most scanned blank type at all sites except Grotta di Fumane (Fig. 6a), where almost exclusively blades and bladelets were scanned for 3DGM studies^31,110^. For the other sites, scanning involved the digitization of blanks from different phases of the reduction sequence, particularly key initialization and maintenance operations. This means that certain blanks were scanned to create a reference collection of meaningful pieces for scientific presentations and teaching purposes. For tools, the proportion of retouched bladelets is notably high at both Grotta di Castelcivita and Grotta di Fumane (Fig. 6b). These differences should not be interpreted as remarkable technological differences between the sites, but rather as a result of the specific scientific objectives during the development of the OAP. It is worth noting, however, that the number of retouched bladelets recovered at Grotta della Cala is extremely low^112^, and that flake production was a significant component of the lithic production at Riparo Bombrini, particularly with the use of low-quality local chert^89,105^. Finally, most of the scanned blanks (Table 3) and tools (Table 4) are complete artifacts, with only a limited number of broken blanks, as 3DGM analyses and other quantitative assessments cannot be performed on broken pieces.

**Table 3:** Distribution of scanned blanks, showing the number of complete and broken (i.e., proximal, mesial, and distal) artifacts at each site.

Site	Complete	Proximal	Mesial	Distal	Total
Grotta della Cala	20 (83.3%)	3 (12.5%)	1 (4.2%)	0 (0.0%)	24
Grotta di Castelcivita	82 (89.1%)	3 (3.3%)	1 (1.1%)	6 (6.5%)	92
Grotta di Fumane	578 (94.6%)	28 (4.6%)	1 (0.2%)	4 (0.7%)	611
Riparo Bombrini	4 (80.0%)	1 (20.0%)	0 (0.0%)	0 (0.0%)	5
Total	684 (93.4%)	35 (4.8%)	3 (0.4%)	10 (1.4%)	732

Percentages are reported in brackets.

**Table 4:** Distribution of scanned tools, displaying the number of complete and broken (i.e., proximal, mesial, and distal) artifacts at each site.

Site	Complete	Proximal	Mesial	Distal	Other/Undet.	Total
Grotta della Cala	90 (62.9%)	9 (6.3%)	5 (3.5%)	32 (22.4%)	7 (4.9%)	143
Grotta di Castelcivita	178 (80.9%)	6 (2.7%)	4 (1.8%)	30 (13.6%)	2 (0.9%)	220
Grotta di Fumane	126 (92.6%)	7 (5.1%)	0 (0.0%)	3 (2.2%)	0 (0.0%)	136
Riparo Bombrini	14 (50.0%)	3 (10.7%)	0 (0.0%)	8 (28.6%)	3 (10.7%)	28
Total	408 (77.4%)	25 (4.7%)	9 (1.7%)	73 (13.9%)	12 (2.3%)	527

The Other/Undet. (i.e., Undetermined) category includes tools made on blanks with undetermined breakage and tools made from other lithic classes, such as pebbles. Percentages are reported in brackets.

## Data Records

### Structure of the repositories

The OAP is organized into four open-access repositories, published on Zenodo (see Fig. 1), with updates and new releases posted on the project website (https://www.armandofalcucci.com/project/open_aurignacian/):

1. **Grotta di Fumane**^4^: 10.5281/zenodo.6362149
2. **Grotta di Castelcivita**^1^: 10.5281/zenodo.10631389
3. **Grotta della Cala**^2^: 10.5281/zenodo.14165189
4. **Riparo Bombrini**^3^: 10.5281/zenodo.14731694

The decision to create four separate repositories was made to ensure proper attribution to the fieldwork director(s) for each site. Each Zenodo repository contains all the necessary data and documentation to ensure proper use. We organized and described these repositories following the guidelines outlined in the book *3D Data, Creation to Curation: Community Standards for 3D Data Preservation*, edited by J. Moore, A. Rountrey, and H. Scates Kettler^126^. Specifically, we prepared a README text file and a CSV (comma-separated values) file based on the example provided in Chapter 2 (“Best Practices for 3D Data Preservation”), which also describes the lithic repository from the Early Upper Paleolithic site of Tvarožná X, Czech Republic, published in the University of Minnesota’s Data Repository (DRUM) by Tostevin *et al*.^125^. The README file provides an overview of the repository and a detailed description of the associated metadata. The CSV file includes several columns documenting the artifacts’ stratigraphic provenience, a summary of each 3D mesh file, and information about data collection and post-processing procedures. Finally, the zipped folder contains the 3D models in PLY (Polygon File Format), which can be opened with various software tools commonly used in lithic analysis, such as Meshlab^29^, CloudCompare (https://www.danielgm.net/cc/), and Artifact3-D^27^. For Grotta di Fumane, a zipped folder also contains meshes in WRL (Virtual Reality Modeling Language) format, available only for selected models (i.e., blades and bladelets) and on a previous version (2.1.1) of the dataset: https://zenodo.org/doi/10.5281/zenodo.7664308. This format was required to run 3DGM analyses of blades and bladelets in the AGMT3-D^28^ software. The size of the zipped folders with the 3D meshes ranges from 380 MB to 1.8 GB. Each Zenodo repository includes an overview section with a general description of the site, as well as a description of the dataset, research and usage notes, licensing information, citation guidelines, and relevant references. Future updates and new versions of the datasets will be published in new versions of the same repository, ensuring seamless reusability and access to the most current data.

### Metadata CSV file

The metadata CSV file included in each repository provides contextual and technical information intended to support the effective use of the associated 3D meshes. Each mesh is identified by a unique identifier (ID), which corresponds to the mesh file name. This identifier is consistent with those used in previously published studies related to the respective sites, ensuring continuity across publications. The CSV file presents a selected subset of attributes for each model. Users seeking more comprehensive techno-typological data can download the relevant research compendia associated with each site (see Table 1). Additionally, the project website (https://www.armandofalcucci.com/project/open_aurignacian/) is regularly updated with links to publications that utilize the 3D datasets, thereby facilitating long-term data reuse.

Table 5 provides a complete list of the stratigraphic, technological, scanning, and post-processing attributes recorded for each model. Technological data were documented by one of the authors (AF) using a standardized attribute analysis system^127,128^, which enabled the collection of both metric and discrete variables. In addition to qualitative attributes, quantitative metrics such as volume (in mm³) and surface area (in mm²) were calculated using the *Rvcg*^129^ package in R^130^. For cores and core-tools, technological classifications follow the system established by Falcucci and Peresani^131^.

**Table 5:** Attributes and data recorded for each 3D scanned lithic artifact, with descriptions of each dataset column.

Attribute	Details
ID	Unique identifier for each 3D model.
Site	The archaeological site where the lithic was excavated.
Layer/Sub-layer	The stratigraphic origin of the lithic.
Raw_material	Categorization by type of raw material (e.g., chert, jasper, quartzite, limestone). In the case of Grotta di Fumane, a more detailed classification (i.e., the name of the geological formation) is provided.
Class	Broad artifact sorting (e.g., blank, core, core-tool, tool), following common classifications in lithic analysis. Cores are artifacts of any size that lack a dorsal/ventral surface but have two or more blade/bladelet/flake scars^128,144^. Tools are artifacts of any size that exhibit retouch along the margins. Core-tools are artifacts that have produced bladelets but can also be classified as tools (e.g., carinated endscrapers or burin cores) following a typological classification^145^. Blanks are flaked artifacts with both a dorsal and ventral face.
Blank	Classification of the blank into flake, blade, and bladelet categories. A blade is defined as a flaked blank whose length is at least twice its width, regardless of shape. Bladelets are defined as blades whose maximum width is less than 12 mm^146^. The other category includes non-chipped blanks such as pebbles.
Technology	Technological classification of the blanks into categories such as initialization, maintenance, optimal, semi-cortical, and other, following Falcucci *et al*.^147^ and Falcucci *et al*.^95^
Core_classification	Technological categories for cores and core-tools (e.g., carinated, multi-platform, narrow-sided, semi-circumferential) following Falcucci & Peresani^131^.
Cortex	Percentage of cortex coverage (i.e., 0%, 1–33%, 33–66%, 66–99%, 100%), estimated visually.
Preservation	Breakage classification for blanks (i.e., complete, distal, mesial, proximal, and undetermined). For cores and most core-tools, preservation is marked as “other”, due to undetermined breakage.
Volume	The volume of the artifact in cubic millimeters.
Surface	The surface area of the artifact in square millimeters.
Length	Maximum length in millimeters based on technological orientation, recorded with a digital caliper.
Width	Maximum width in millimeters based on technological orientation, recorded with a digital caliper.
Thickness	Maximum thickness in millimeters based on technological orientation, recorded with a digital caliper.
File_list	The list of files in the dataset that correspond to this specific ID.
Model_unit	The unit of measurement used for the 3D model. When viewing the artifact in a 3D viewer that supports real-world units, this is the unit you enter into your program to ensure proper scaling. Note that this is not related to the object’s resolution, but is the value needed for accurate scaling when importing the model into your 3D program.
#_of_polygons	The number of polygons (or faces) in the 3D model of the artifact.
Avg_edge_length(mm)/Resolution	The average distance (in mm) between points on the 3D model, serving as an effective measure of the model’s resolution. Avg stands for average.
Resolution_score	A value assigned to each model, reflecting its resolution. Drawing from the full set of scans from the Open Aurignacian Project, it categorizes artifacts into four groups (i.e., ultra-detailed, detailed, moderate detail, low detail) according to their average edge length value, offering a qualitative evaluation of the model’s resolution in comparison to others in the project.
Scanner	The specific model of the scanner used to capture the 3D data of the lithic artifact (i.e., Artec Micro 1, Artec Spider 1, and micro-CT).
Scan_software	The version of the software used in conjunction with the scanner to capture the 3D data of the artifact.
Postprocessing_software	The version of the software used to execute postprocessing algorithms and generate the final 3D mesh of the artifact.
Coating	Yes/No entry specifying if coating was used for any scan.

## Technical Validation

All datasets and 3D models presented in this study have already been utilized in scientific investigations, addressing several technologically and behaviorally relevant questions. This prior use serves as a valuable validation of their accuracy and relevance for archaeological research. The Artec Spider and Artec Micro scanners were properly calibrated to ensure accuracy and underwent frequent check-ups and inspections by the manufacturer to verify their precision. The scanning process involved capturing the lithics from different angles to record all surfaces and edges. Post-processing algorithms, recommended by Artec Inc., were applied to align the scans, fill holes, remove outliers, reduce the number of polygons to optimize model size while maintaining sufficient resolution, and finally export the 3D mesh in PLY format. For the micro-CT, the accuracy of the scans was validated through comparison with high-resolution models obtained from the Artec Micro^19^. This comparison confirmed that the micro-CT scans provided an adequate level of precision for archaeological investigations, ensuring the validity of the data for further analysis. While every effort was made to ensure the highest quality of scans, it is important to note that the resolution of the micro-CT scans for very fine details was slightly lower than that of the Artec Micro. However, the overall quality and suitability for archaeological purposes is deemed sufficient, especially for larger or more robust artifacts. The step-by-step protocols used to scan all published 3D models were developed by one of the authors (AF) and are available on *protocols.io*^22,124^. The *Styrostone* protocol provides detailed workflows for both micro-CT and Artec Spider scanning: 10.17504/protocols.io.4r3l24d9qg1y/v3. The *Microstone* protocol outlines the workflow for Artec Micro scanning: 10.17504/protocols.io.81wgb6781lpk/v1.

## Usage Notes

To access the datasets, please follow the link associated with each repository and download all data contained within. After unzipping the folders, the meshes can be opened using various 3D visualization programs, such as the open-source Meshlab (https://www.meshlab.net/). The README and CSV files within the dataset can be used to seamlessly select the necessary files for analysis. The data presented in these repositories can be used for a variety of analyses, including 3DGM assessments, reduction intensity studies on both cores and tools, investigations on edge angles for both retouched and unretouched blanks, morphometric and technological analyses on cores, as well as lithic classifications using machine learning algorithms. Additionally, the data can be used to extract other relevant information, such as outlines for 2D geometric morphometrics.

The repositories will undergo future updates and new releases, which may include additional meshes and/or scanned artifacts. As such, the DOI of each repository links to the “cite all versions” option on Zenodo, ensuring that the latest version is always accessed. New releases will be accessible to scholars and other interested parties, with proper version labeling in accordance with the FAIR principles. We encourage researchers using these datasets to cite both the Zenodo repository and this paper.

## Data Availability

The dataset and R scripts used to present and visualize the structure of the four datasets are available on Zenodo^132^: 10.5281/zenodo.15131493. All data presentation and visualization steps were performed in R v.4.3.1^130^ and R Studio^133^ under Windows 10, utilizing the *Tidyverse* packages^134^ and *ggstatsplot*^135^. The merged datasets from the four sites are provided as a supplementary file to facilitate smooth searching of the required data.
